# Hybrid neurofibroma/schwannoma in schwannomatosis—a diagnostically challenging benign peripheral nerve sheath tumour

**DOI:** 10.1007/s10689-025-00505-4

**Published:** 2025-11-17

**Authors:** Daniel Tippner, Maxim Anokhin, Jonas Scheffler, Fabio Hellmann, Stefan K. Plontke, Sandra Leisz, Anja Harder

**Affiliations:** 1 CURE-NF Research Group, Medical Faculty, Martin Luther University Halle-Wittenberg, Große Steinstraße 52, 0611 Halle (Saale), Germany; 2 Department of Otorhinolaryngology, Head & Neck Surgery, Martin Luther University Halle-Wittenberg, Halle (Saale), Germany; 3 Medical Care Centre, Neurosurgery and Neuroradiology, Gensingen, Germany; 4 Chair for Human-Centered Artificial Intelligence, Faculty of Applied Computer Science, University of Augsburg, Augsburg, Germany; 5 Department of Neurosurgery, University Medicine Halle, Halle (Saale), Germany; 6 Medical Faculty, University of Muenster, Münster, Germany

**Keywords:** Peripheral nerve sheath tumour, Schwannomatosis, SWN, Schwannoma, Hybrid neurofibroma/schwannoma, Neurofibromatosis, NF2, LZTR1, SMARCB1, ERBB2, Molecular profiling, Hybrid

## Abstract

Hybrid neurofibroma/schwannoma tumors (HNS) represent a still underrecognized, yet clinically and diagnostically significant entity within the spectrum of schwannomatosis (SWN). While classical schwannomas have been well known for decades, HNS have only recently been described as a distinct histological pattern, composed of intermixed features typical of both schwannomas and neurofibromas. Differentiating HNS from pure neurofibroma (Nf) is critical, as misclassification may lead to an incorrect diagnosis of neurofibromatosis type 1 rather than SWN. The distinction of hybrid tumors (more precisely HNS) is especially important in SWN forms outside the neurofibromatosis type 2 (NF2) spectrum (NF2-SWN), where major diagnostic criteria are less well defined, making histological differentiation even more significant. At the molecular level, HNS frequently show alterations in the genes *NF2*, *LZTR1*, and *SMARCB1*, often accompanied by characteristic losses of chromosome 22q. In addition, recurrent somatic mutations have been identified in genes such as *ERBB2*, *RET*, *KMT2A*, and *CTNNA3*. Methylation profiling classifies HNS within the schwannoma spectrum, supporting the hypothesis that they may be a morphological variant rather than a distinct entity, although this has not yet been conclusively confirmed. Histologically, HNS are characterized by a combination of mostly schwannoma-associated Antoni A patterns, collagen-rich neurofibroma-like areas, lymphocytic infiltrates, and, in some cases, plexiform growth. Given the diagnostic challenges, artificial intelligence-based image analysis, such as whole-slide imaging and radiomics, may offer valuable tools for more accurate identification of these tumors in the future. Initial studies in related fields have shown that such approaches can even surpass human-level accuracy. Nevertheless, an accurate histological and, if necessary, molecular evaluation remains essential—particularly for the correct classification as SWN and for ensuring appropriate genetic counseling to affected individuals.

## Introduction

Peripheral nerve sheath tumours (PNST) in schwannomatosis (SWN) comprise schwannoma (Sw) and hybrid neurofibroma/schwannoma (HNS). The second tumour type, the hybrid variant, is recognized on the basis of relatively recent insights into the neuropathological characterization of SWN-associated neoplasms. However, it is ONLY as recent as the updated diagnostic criteria proposing the term “schwannomatosis” as an umbrella term for neurofibromatosis type 2 (NF2) and SWN, that novel classification of SWN based on disease-causing genes was proposed [1]. It is important to know this history to correctly interpret publications before 2022 that differentiated between NF2 and SWN.

The 2022 international consensus recommendation correctly pointed out that misdiagnosis of Sw and HNS as neurofibroma (Nf) has led to numerous incorrect diagnoses of neurofibromatosis type 1 (NF1). Accurate diagnosis is particularly important in forms of SWN not related to *NF2* variants, such as *LZTR1*- or *SMARCB1*-associated SWN, or SWN-NEC (not elsewhere classified), as these subtypes are defined by fewer major diagnostic criteria compared with NF2-related SWN. Consequently, the histopathological identification of Sw or HNS plays a significantly more critical role in the diagnostic process and has thus been explicitly included in the official recommendation tables.

HNS have been reported in a few studies so far. It took 80 years (Sw was first recognized histologically in 1910 by Jose Verocay) for HNS to be distinguished from conventional Sw by Feany and co-workers, who described a series of 9 cases in 1998 [2]. In 2012, a clear association of HNS to neurofibromatosis type 1 and especially to SWN (certainly back then NF2-SWN and SWN-NEC) was described by Harder and co-workers, who also first elucidated the molecular genetics in a subsequent study [3, 4]. Thereafter HNS was integrated into tumour WHO classifications [5, 6]. In 2016 another detailed histological study of HNS in 11 cases showed clear evidence of occurrence in NF2-SWN [7]. New molecular techniques allowed a more comprehensive routine mutation screening in these benign tumours and led to the detection of additional genetic events, such as variants in *ERBB2* associated with mosaic SWN and responsive to lapatinib [8]. More recently a role for variants of *KMT2* was uncovered in a rare intracerebral HNS [9]. The updated classification of SWN by Plotkin and co-workers in 2022 has shed new light on these tumours and their genetics [1].

Strictly speaking, the term ‘hybrid nerve sheath tumour’ is not precise. It is a collective term for different types of nerve sheath tumours that consist of components that are historically and conventionally assigned to one or another typical nerve sheath tumour. For SWN, only the HNS has a major role. With the introduction of the diagnosis of HNS into the different WHO classifications of tumours, this diagnosis should be made more frequently. But pathologists’ awareness is clearly limited. This is because (1) HNS is a benign tumour that is usually diagnosed without molecular analysis; (2) the variable proportion of the two different components can obscure one of them (which is why an Nf or an Sw is more likely to be diagnosed); and (3) the differential diagnosis may be difficult.

Take home messages

- The schwannoma (Sw) and hybrid neurofibroma/schwannoma (HNS) are the characteristic peripheral nerve sheath tumours (PNST) in schwannomatosis (SWN).
- HNS is a subtype of hybrid PNST that can easily be misdiagnosed.
- Wrong classification of HNS as neurofibroma (Nf) can lead to misdiagnosis (instead of potentially associated SWN, the completely different neurofibromatosis type 1 might wrongly be suggested).

### Conventional diagnosis of hybrid neurofibroma/schwannoma

Schwannomas (Sw) are the most frequent tumours among benign PNST and are subdivided into conventional/classical, cellular, epithelioid, plexiform, and melanotic. The latter is typical for patients with Carney complex and *PRKAR1A* variants and not supposed to be typical in other hereditary conditions. Sw can be localised peripherally, viscerally, intraspinally, and intracranially. Sw can also be localised to very particular locations, as in the case of rare inner ear schwannomas (IES) [10]. Most IES cases appear to be sporadic, although a significant proportion are associated with NF2-related SWN [11, 12]. So far there are no reports of HNS in the inner ear. Given the phenotypic overlap between *NF2-* and *SMARCB1/LZTR1*-related SWN along with the potential contribution of mosaicism, further genetic studies are warranted in patients with IES [1, 13, 14].

The histological pattern of Sw comprises medium-sized, spindle-like tumour cells with long bipolar extensions and oval, elongated, or even roundish nuclei and arrangement in a fishlike pattern. Palisade positions of the tumour cell nuclei with alternating nucleus-rich and nucleus-poor being recognized as pathognomonic by Jose Verocay in 1910 and are referred to as Verocay bodies after him. The dense cellular pattern is referred to as Antoni type A (fibrillar type) after the Swedish physician Nils Ragnar Eugene Antoni. A tumour containing hypocellular, myxoid, or loosened accumulations of histiocyte-like, vacuolated, or fatty Schwann cells, is referred to as Antoni type B. A balanced ratio of Antoni type A and B areas is called conventional Sw. In the stromal part, thick-walled blood vessels with stores of hyaline material in the vessel walls are noticeable. Sometimes cavernoma-like vessels are formed. Fresh and older haemorrhages and abundant perivascular haemosiderophages are very typical. A schwannoma with regressive changes (so-called ‘ancient Sw’) demonstrates collagen deposits, fibrin thrombi, and pseudocystic regressions as well as a considerable nuclear pleomorphism and anisomorphism, which was formerly classified as a ‘degenerative polymorphic pattern’ and is not an anaplastic feature. A tumour with significantly increased cellularity and presence of a fascicular, storiform, or even non-characteristic picture is classified as a cellular Sw. Plexiform Sw offer a special growth pattern involving several nerve fascicles and intraneural growth. Despite various special forms, Sw are basically benign tumours that are classified as grade I according to the WHO classifications, although melanotic psammomatous Sw's are associated with a less favourable prognosis. Immunohistochemically, Sw shows an almost continuous positive immune reaction for the S100beta protein, with cytoplasm and nuclei being labelled. Furthermore, collagen IV marks the basement membranes of Schwann cells, which can also be visualised with silver (e.g., reticulin) staining. Experience has shown that the growth fraction, measured by the Ki67 staining index, is 2% to 5%, but can reach values of up to 15% in foci, particularly in cellular schwannomas. The loss of expression of *LZTR1* and *SMARCB1* may indicate SWN, although there is often only a partial loss, a ‘mosaic pattern’ [15]. Experienced neuropathologists cannot tell from histology alone whether a Sw occurs sporadically, singly, or multiply as part of a tumour syndrome or where it is localised in the body. The same applies to an Nf. However, if a plexiform structure is present or has been preserved by the operation, then a tumour syndrome can already be assumed, although there seem to be exceptions too (both plexiform Sw and Nf can appear to be sporadic although both may be due to very localised mosaicism) [15, 16].

Neurofibromas (Nf) originate from precursor cells of Schwann cells and are proliferations of both tumour cells (*NF1*^−/−^) sharing characteristics with unmyelinated Schwann cells and non-tumour Schwann cells (*NF1*^+/−^). They additionally contain nerve fibres, fibroblasts, macrophages, mast cells, and perineural cells interspersed with collagen, often within thick bundles. The Schwann cells display chromatin-dense wavy nuclei. Cytologic atypia or mitotic figures are typically absent in the benign tumour present in HNS. The tumour cells are embedded within a basophilic extracellular matrix enriched in glycosaminoglycans and collagen fibres. Plexiform growth is common in NF1. Immunohistochemically, partial immunoreactivity for S100 protein represents the proportion of Schwann cells. However, the number of S100 protein–positive cells is significantly lower compared with that in Sw. Additional focal immune-reactivities may be observed for glial fibrillary acidic protein (GFAP), vimentin, CD34, and factor XIIIa.

**Fig. 1:**
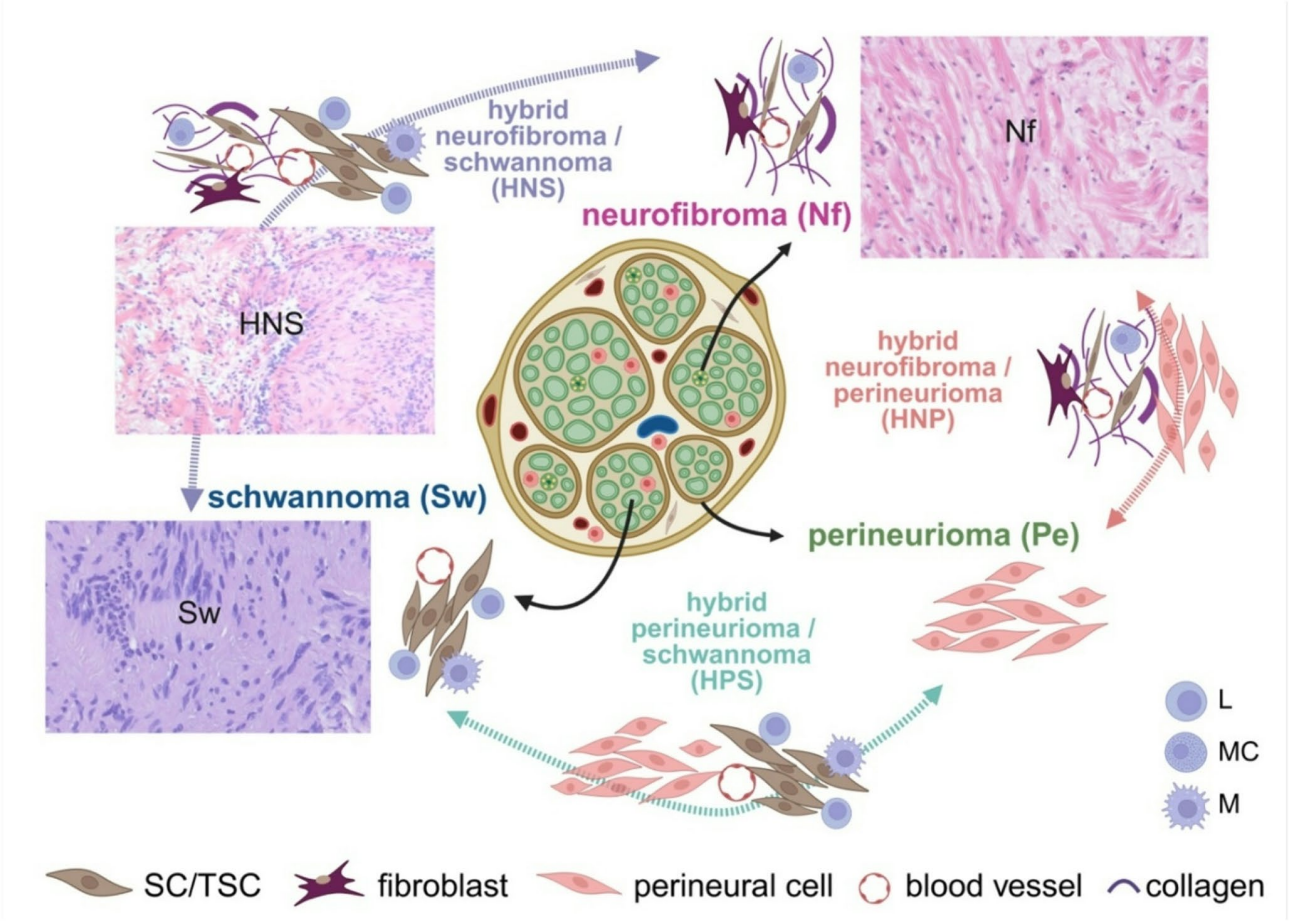
Benign hybrid peripheral nerve sheath tumours and their conventional tumours of origin. Although current studies document greater variability in the cellular tumour components and their origins, from a purely histological point of view schwannomas (Sw) are still supposed to share most characteristics with myelinating Schwann cells, neurofibromas (Nf) with non-myelinating Schwann cells, and perineuriomas (Pe) with perineural cells (indicated by the arrows originating from the appropriate structure of a normal nerve). The hybrid variants show histological characteristics of two different nerve sheath tumours and are here depicted between the classical entities (variant hybrid neurofibroma/schwannoma/perineurioma is not shown). SC, Schwann cells; TSC, tumour Schwann cells; L, lymphocytes; MC, mast cells; M, macrophages. As this review is mainly concerned with Sw, HNS, and Nf, typical histological patterns are shown (the photos are of tumours from previous studies [3, 4]). Created in BioRender. Leisz, S. (2025); agreement number: VH28JQMTK8; https://BioRender.com/iugbu4k

A hybrid peripheral nerve sheath tumour (HPNST) is a primarily benign neoplasm consisting of combinations of Nf, Sw, or perineuriomas (Pe) (Fig. 1). HPNST were first recognised in the WHO classification in 2013 [5, 6, 17]; the exact percentage of NST that are HPNST is not yet known. A recent review demonstrated that schwannoma/perineurioma is the most frequent type and is mainly sporadic [18, 19]. Although comprehensive studies are lacking, HNS are reported to constitute about 19% of HPNST [20]. Concerning HNS, to date only three larger studies have systematically analysed HNS tumour series, and all of them have demonstrated an association of HNS with tumour suppressor gene syndromes, especially SWN [2, 4, 7]. Salzano and co-workers have also recently reviewed some case reports [20]. Occurrence of HNS was rarely reported in NF1 (9%) but can be questioned looking back as these data were retrieved from submission pathology forms probably not all having been checked by geneticists; nevertheless, HNS might still occur in NF1, although they are increasingly reported in SWN [4]. In SWN, Sw and HNS occur at the identical locations involving nerves, mostly at peripheral or spinal nerves, but rare intracranial manifestations at the olfactory groove and intramuscular, intraorbital, and intraoral localizations are reported [9, 21–23].

HNS consist of two histological intermingled growth patterns—those of typical Sw and of Nf, in varying percentages. Sw-like areas are mostly Antoni A regions; Antoni B regions may also occur, but only in large tumours. As typical for Nf, Nf-like areas often include abundant collagen deposits, myxoid changes, and fragments of neurofilament-positive, broken nerve structures. Nf-like areas are characterised by elongated and wavy-appearing tumour cells as seen in classical Nf. Alike in Sw and Nf, HNS can demonstrate a plexiform growth pattern, which was seen in nearly 30% of cases in one series [4]. In that series, it was also remarkable that Nf-like areas always surrounded the Sw-like nodules and that the Sw-like nodules were sharply encapsulated by collagen bundles from the surrounding Nf-like tissue. Many features typical of Sw are recapitulated in HNS, such as pronounced lymphocytic infiltrates. These Schwann cell–derived HNS express S100beta, which is dense in the schwannomatous component. The Ki-67 index is 0.8–18.5%. Until now, only one HNS has been described to show premalignant alterations with multiple chromosomal imbalances [3]. In a recent study, the Nf- and Sw-like HNS components were associated with distinct spatial gene expression clusters and transcriptional programmes [24]. Moreover, expression of APOD was proposed as a potential discriminator between these two components in seven follow-up cases. However, since the same study also demonstrated APOD expression in Antoni B areas of Sw and was based on only two tumours, further investigations meeting the criteria for a statistically robust power analysis are required to validate these findings. Nonetheless, this highlights the importance of distinguishing Antoni B areas from Nf-like regions of HNS in the diagnostic process.

To summarize, the required main criteria for a neuropathological diagnosis of an HNS include (1) occurrence of both Sw and Nf in the same lesion; (2) complete fulfilment by those areas typical of a Nf of the criteria for an Nf (grade I CNS according to the current WHO classification) for example, pronounced collagen deposition; and (3) complete fulfilment by areas typical of Sw of the criteria for Antoni A regions of an Sw (grade I of the current WHO CNS classification). On the basis of our experience, we suggest that at least 15% of the total HNS area should consist of either neurofibroma (Nf) or schwannoma (Sw) components to allow for reliable histological identification of both. Smaller proportions may be diagnostically challenging. In such cases, the tumour should be recut macroscopically and embedded more extensively to improve diagnostic accuracy. One should also check carefully that areas typical of Nf do not mimic Antoni B areas, by looking for evidence of collagen fibres and a mixture of Schwann cells and fibroblasts. One should be alert to tumours appearing with a plexiform growth pattern. Typical pronounced lymphocytic infiltrates and a sharp demarcation between the Nf and Sw components can be helpful minor diagnostic criteria.

**Fig. 2:**
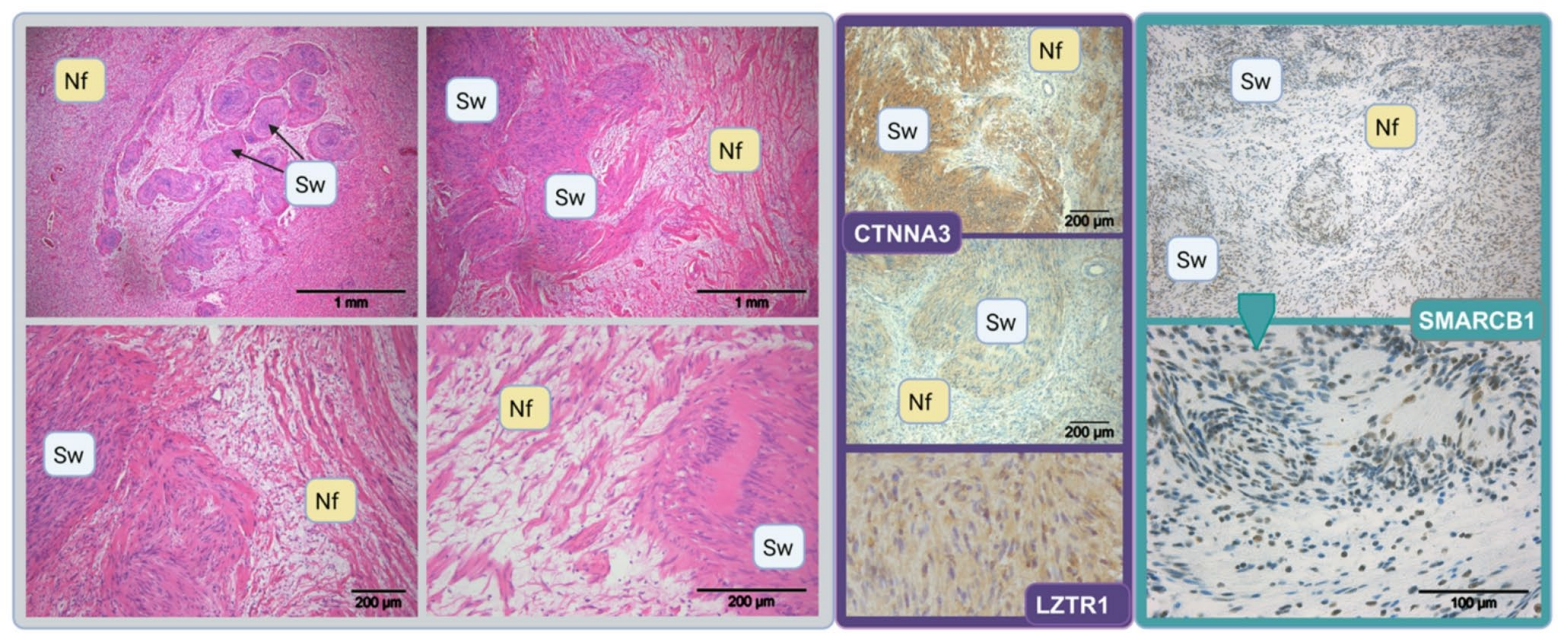
Typical histological pattern of hybrid neurofibroma/schwannoma (HNS). Right panel: Four different haematoxylin and eosin (H&E)–stained slides demonstrating the side-by-side combination of histological features characteristic of both neurofibroma (Nf) and schwannoma (Sw). Middle panel: CTNNA3 expression can be lower than normal in some HNS (compare upper picture with normal expression to picture below with reduced expression). In *LZTR1*-SWN, expression of the *LZTR1* protein is lost (below) in nearly all nuclei. Left panel: In *SMARCB1*-SWN, a mosaic pattern of *SMARCB1*-positive and negative nuclei is detected. Photos are of tumours from previous studies [3, 4]. Created in BioRender. Lohse, S. (2025); agreement number: ZN28JQN57J; https://BioRender.com/a2zc7k7

SWN-related hybrid tumours are characterized by a typical mosaic staining pattern of protein *SMARCB1* (positive and negative nuclei) and a remarkable loss of LZTR1 (only few positive nuclei left) in LZTR1-SWN. Involvement of other proteins of the chromatin remodelling complex has also been investigated [15]. Nevertheless, immunohistochemical staining—for example, for BAF170, NF2 protein merlin or others to show reduced protein expression due to LOH or mutations—might not be safe for routine diagnostic use. Therefore, the observation of important features from just daily routine haematoxylin and eosin (H&E)–stained slides (Fig. 2) is very helpful for the differential diagnosis. Advances in automated analyses based on deep learning could provide a cost-effective and rapid alternative to filtering HNS instead of performing expensive molecular analyses to ensure correct diagnosis.

Finally, new molecular single-cell analyses of Sw reveal that the cellular components are much more complex, with a greater diversity of Schwann cells than previously assumed: loss of the myelinating phenotype, myeloid cell infiltrations, so-called repair-type Schwann cells, and many more Schwann cell subtypes such as Schwann_VEGFA, Schwann_SCN7A, Schwann_PRX, and Schwann_CRLF1, according to their single-cell transcriptional and functional profiles [25, 26]. So far, however, this has not been applicable in achieving a histologically reliable and neuropathologically usable diagnosis.

Malignant transformation to MPNST arising from benign PNST in SWN has been described in a few studies so far even without history of radiation [27–30]. However, there is little reliable knowledge about the processes of malignant transformation in HNS. However, individual descriptions indicate that further molecular changes associated with malignancy may also occur in the HNS [3], meaning that accurate histological diagnosis remains an important tool in everyday routine practice for identifying underlying abnormal patterns and referring them for molecular analysis.

Take home messages

- Diagnosis of an HNS requires occurrence of both Sw-like and Nf-like tumour areas in the same lesion that, respectively, fulfil the criteria for an Nf and an Sw (grade I CNS WHO classification).
- A plexiform growth pattern is common, but every plexiform peripheral nerve sheath tumour should suggest the presence of a tumour syndrome and requires special diagnostic care.
- HNS often show pronounced lymphocytic infiltrates and a sharp demarcation between the Nf and Sw components.

### Molecular diagnosis of a hybrid neurofibroma/schwannoma

Since the correct diagnosis of HNS can be extremely important for the differentiation of NF1 and SWN, molecular diagnostics may be of particular importance in special cases. This was demonstrated in our daily practice in a case in which there were enquiries from the clinical side because the past diagnosis of a Nf did not match the clinical picture in any way. A detailed family history, clinical examination and genetic diagnostics revealed a mutation in the *LZTR1* gene, which led to the re-diagnosis of all the patient's tumours, which turned out to be exclusively HNS at different locations. Nevertheless, it turns out that the number of molecular analyses of HNS at the somatic level is very limited. This may be due to the fact that these tumours are still too rarely diagnosed, and thus no larger series are created that are suitable for high-throughput analyses. Until the contrary is proven, the only question that remains is whether HNS, although they are distinguishable from Sw by histology, represent a separate molecular entity, because they show the molecular changes we see in Sw. The question remains if they are only a morphologic variant of Sw.

The fact that HNS might be a variant of Sw is supported by data from Röhrich et al. [31], who reported that tumour methylation profiles of HNS cluster with those of benign Sw. Sw were described to form four different methylation subgroups (I–IV), and HNS were mostly localized in cluster II. In support of this, two HNS investigated by spatial gene expression analysis were also found to cluster to the Sw methylation class [24]. However, other methylation clusters have been described by Agnithori et al. for Sw [32].

Since only a few molecular data have been collected for HNS, but a great deal for Sw, we first look at Sw, especially since HNS may represent only a subtype of Sw at the molecular level. For the development of Sw in *LZTR1*- and *SMARCB1*-related SWN, a multistep-model involving at least four hits or three steps has been postulated [33, 34]. Knudson’s two-hit model needed to be refined due to a higher complexity concerning genes involved in SWN such as *LZTR1*, *NF2,* and *SMARCB1,* and rarely *SMARCCA4 and COQ6* [35, 36]. A typical and frequent somatic (second) hit (about 61% in all Sw according to Agnithori and co-workers) in Sw is complete loss of chromosome 22 or of 22q, including all or some of these genes located on chromosome 22 (reviewed in detail by Kehrer‑Sawatzki et al.); thus, indicating that at least two different tumour suppressor genes are involved [32, 34]. Beyond *LZTR1*, *NF2*, *SMARCB1*, *SMARCA4*, *COQ6*, more genes have been detected to be involved in Sw pathogenesis. These include genes encoding the chromatin modifiers *ARID1A/B*, *DDR1*, *TSC1/2*, and a recurrent *SH3PXD2A:HTRA1* fusion (balanced translocation on 10q) and some other rare [32, 37]. Interestingly, *CDKN2A* germline variants have been detected in SWN patients with melanoma as well as PNST that seem to match HNS, and *DGCR8* variants have been detected in SWN patients with euthyroid multinodular goiter [37–39]. Albeit not in this abundance, HNS have been demonstrated to show involvement of nearly the same set of genes and frequent monosomy 22 [3].

**Fig. 3:**
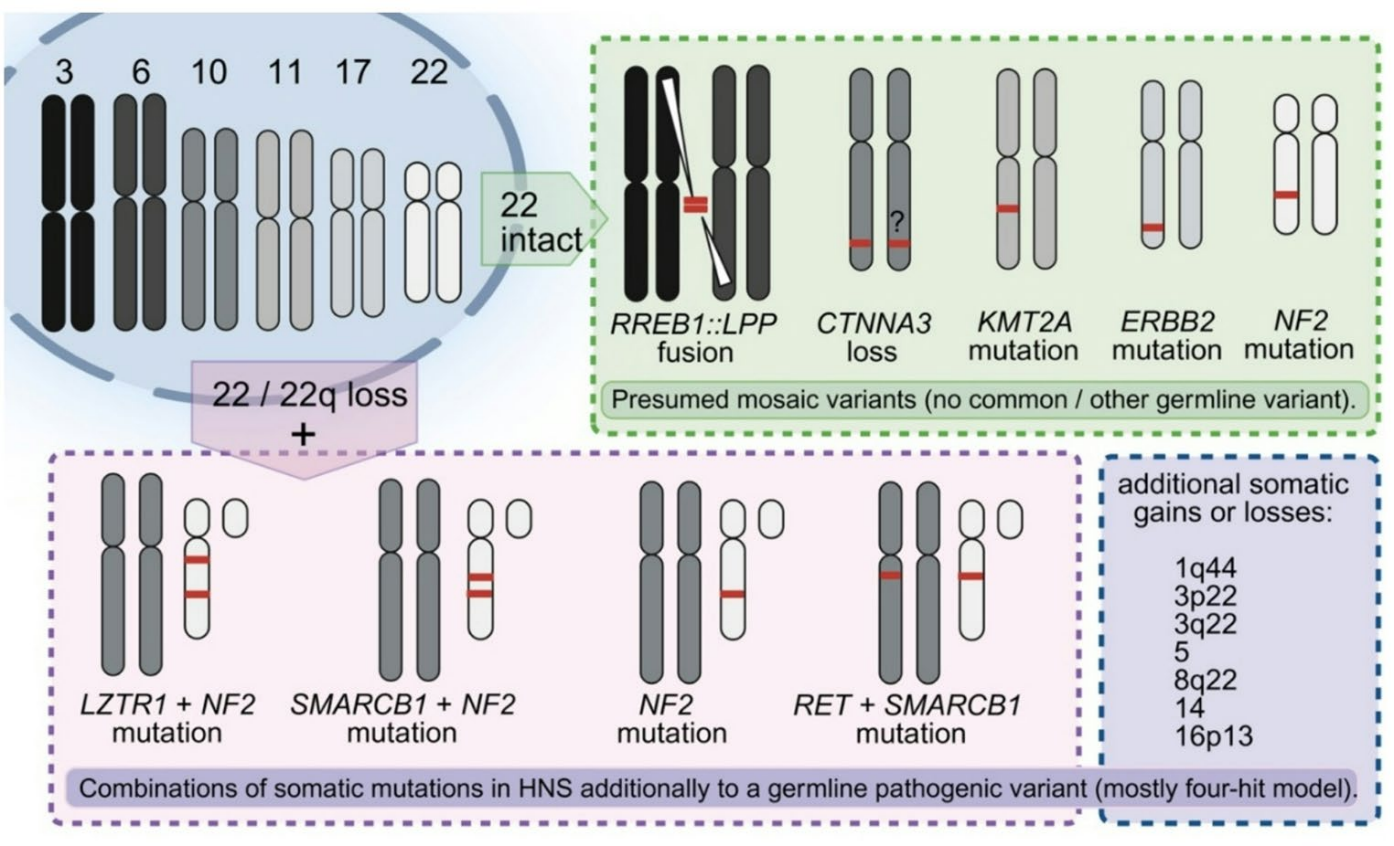
Overview of pathogenic variants in hybrid neurofibroma/schwannoma (HNS). According to the literature, about half of all HNS cases associated with germline SWN show loss of chromosome 22 or 22q additionally to mutations of the genes *NF2* (methylation as epimutation is also possible), *LZTR1*, *SMARCB1*, or *RET*. Additionally, SWN can be associated with a mosaic variant pattern only (no germline mutation) involving genes *ERBB2*, *KMT2*, *NF2*, or fusion *RREB1::LPP*, and might comprise more events that might be increasingly detected as sequencing approaches improve. The *CTNNA3* mutation (shown as presumed somatic) was evaluated to be constitutional; nevertheless, it was not proven in the germline, as only tumour material was investigated. Additional minor chromosomal changes are reported indicating that tumour subtypes might exist. This still leaves the possibility that a transformation to atypical tumours or different risks might occur -as yet, this cannot be safely ruled out. Created in BioRender. Lohse, S. (2025); agreement number: VQ28JQLYGU https://BioRender.com/3qtskh2

In general, genetic variants in HNS comprise germline and somatic events that are also detected in Sw in genes such as *NF2*, *LZTR1*, and *SMARCB1*. These genetic variants need to be differentiated and might help to define a genetic disease such as SWN if the germline mutations are confirmed (e.g., from blood cells). Detection of pure somatic pathogenic variants indicates mosaicism or postzygotic events that occur after the fusion of the male and female germ cells to form the zygote, as shown for somatic variants of *ERBB2*, *RET,* and *KMT2A* and for a rare gene fusion *RREB1::LPP *[8, 9, 19]. It is not known if specific somatic variants are associated with specific tumour localization; one might suspect this of specific manifestations such as the rare intracranial HNS occurrence described in a single report [9]. The recent study by Goto and co-authors identified a somatic *KMT2A* variant, c.4408C > T, p.Q1470*, and trisomies (chromosomes 5 and 14q) while other gene variants (*NF1*, *NF2*, chr. 22 loss), especially germline mutations, were excluded. Furthermore, in one of 22 cases, Stahn et al. reported a focal constitutional deletion of *CTNNA3* located at chromosome 10q21.3 [3]. In addition, transient knockdown of *CTNNA3* in Schwann cells resulted in cytoskeletal alterations and reduced E-cadherin expression, indicating aberrations of epithelial–mesenchymal transition (EMT)-like processes. The utmost recurrent event is a heterozygous deletion of chromosome 22 (monosomy) or at least 44% of 22q that varies within the studies. However, to date, besides a limited transcriptomic approach of two cases [24], the molecular characteristics of HNS have been investigated only in very small case series by Harder et al., Goto et al., and Ronellenfitsch et al. [4, 8, 9], whose main findings are summarized in Table 1 and Fig. 3.

**Table 1:** Summary of genetic alterations in SWN-associated HNS (NF2-related or non-NF2-related)

Gene	Alteration	Somatic Mutation Pattern	References
*22* or *22q*	heterozygous deletion	In part combined with mutations in *LZTR1*, *NF2*, *SMARCB1*, *RET* and chromosomal abnormalities	Ronellenfitsch et al. [8] and Stahn et al. [3]
*NF2*	c.955C > T c.817_824del c.623T > C c.491delC c.561delA c.1145-1149insT c.576C > G gene deletion	Combined with *SMARCB1* mutation(same mae at the same level as c.955C > T) Combined with *SMARCB1* mutation(at the same level as c.623T>C) Combined with *LZTR1* mutation(at the same level as c.1145-1149insT)	Ronellenfitsch et al. [8] and Stahn et al. [3 ]
*ERBB2*	c.2305G > T c.2264T > C c.2329G > T	Activating mutations, no other variants detected so far	Ronellenfitsch et al. [8]
*KMT2A*	c.4408C > T	Combined with chromosomal abnormalities	Goto et al. [9]
*SMARCB1*	c.1130G > A	Combined with *NF2* mutation	Ronellenfitsch et al. [8]
*LTZR1*	c.614T > C	Combined with *NF2* mutation in germline *NF2*	Ronellenfitsch et al. [8]
*RET*	c.2372A > T	Activating mutation. Combined with *SMARCB1* mutation	Ronellenfitsch et al. [8]
*CTNNA3*	deletion	Presumably constitutional	Stahn et al. [3]
*RREB1*	*RREB1::LPP*	Structural gene rearrangement / gene fusion	Dehner et al. [19]

Although there is increasing evidence that HNS are more like Sw in molecular terms and are typical of SWN, *NF1* variants should always be investigated and ruled out for safety reasons to prove the contrary (as there are also reports of HNS in NF1) if the previous diagnosis and molecular data are inconclusive. Detection of mosaics is becoming increasingly important in this context.

Furthermore, hybrid schwannoma/perineurioma has recently been shown to harbour recurrent gene fusions, most common *VGLL3* rearrangements. This supports the hypothesis that these tumours represent a distinct subgroup and not a morphologic variant [19, 40]. In contrast, no recurrent genetic alteration was seen in investigated HNS (n = 3) in this study; leaving it molecularly unproven whether HNS is a separate entity or, for example, a special variant of a Sw.

**Fig. 4:**
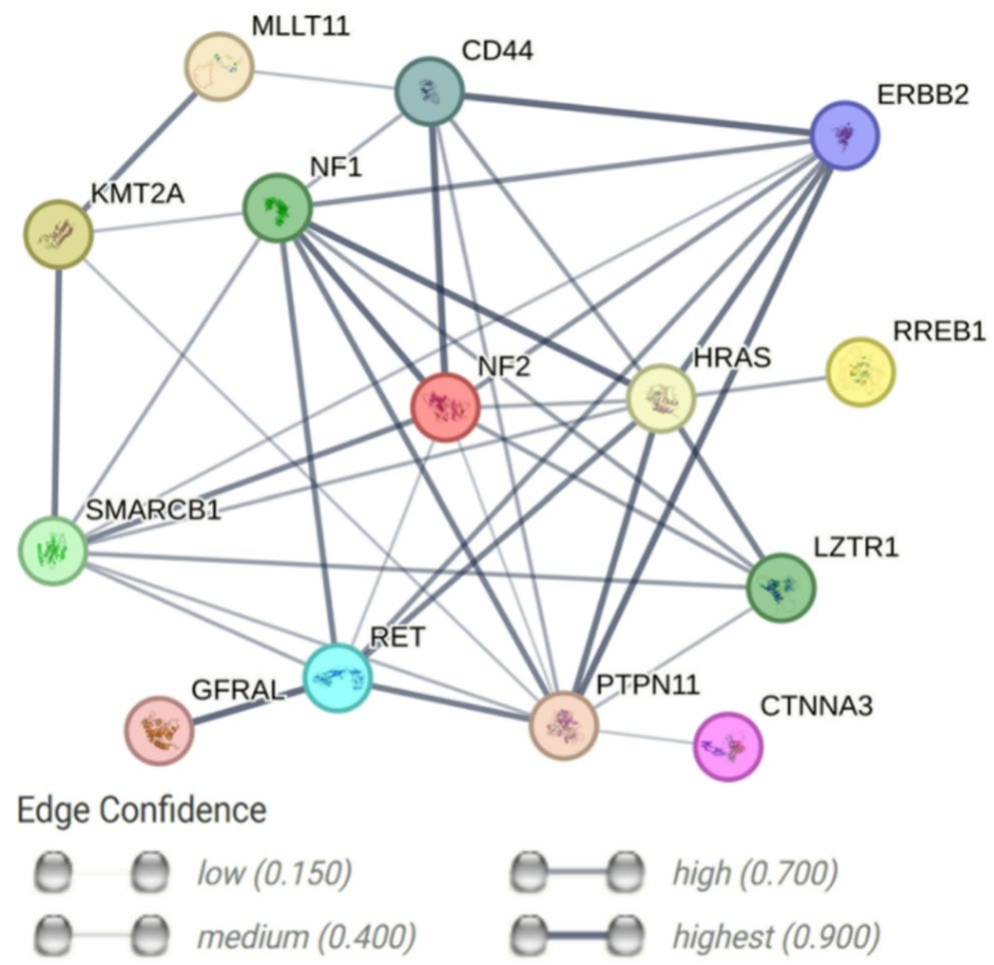
Protein interaction network of players in hybrid neurofibroma/schwannoma known to date. The network nodes represent proteins, while the edges indicate protein–protein associations. These interactions encompass both direct (physical) and indirect (functional) relationships, based on known and predicted protein–protein interactions. Data were obtained from STRING database version 12.0 on 24/07/2025 [41]. GFRAL, GDNF family receptor alpha-like; PTPN11, tyrosine-protein phosphatase non-receptor type 11

An analysis of the genes known to be affected in HNS using the Search Tool for the Retrieval of Interacting Genes/Proteins (STRING) database [41] revealed that the majority of these genes interact functionally or directly with the highest confidence level (Fig. 4). Genes encoding other proteins such as GDNF family receptor alpha like (GFRAL) and Tyrosine-protein phosphatase non-receptor type 11 (PTPN11) could also play a role here due to the high level of interaction in this network.

**Fig. 5:**
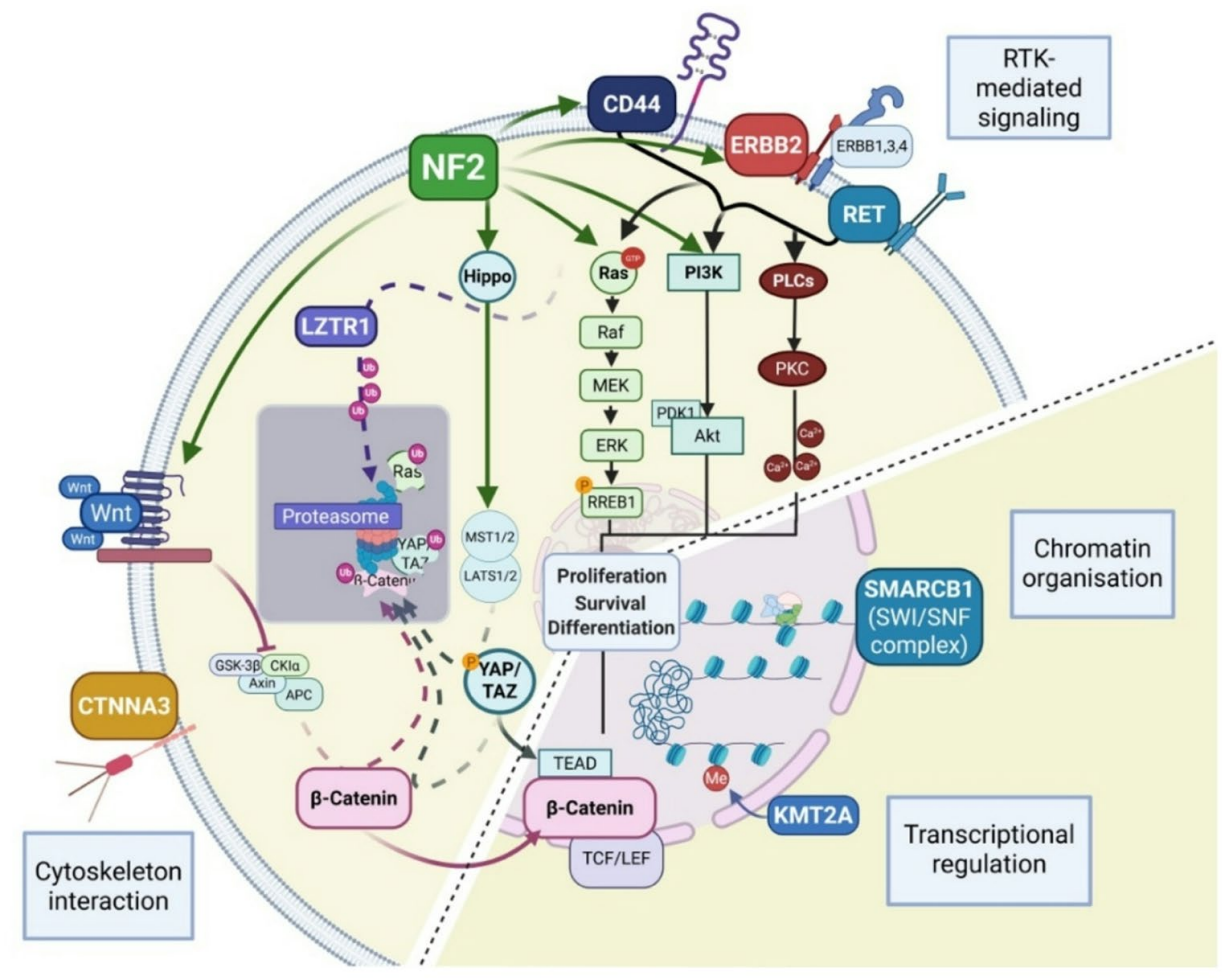
Molecular mechanisms and signaling pathways described for HNS in SWN. The oncogenes *RET* and *ERBB2* encode receptor tyrosine kinases (RTKs) that activate downstream signaling via the Ras, PI3K, and PLCγ pathways, promoting cell proliferation, differentiation, and survival [8, 42]. Merlin, encoded by the *NF2* gene, acts as a tumor suppressor by regulating RTK signaling, cytoskeletal organization, and cell-to-cell contact [43]. LZTR1 functions as an adaptor protein within the Cullin3 ubiquitin ligase complex, facilitating the ubiquitination and subsequent proteasomal degradation of Ras proteins [44]. SMARCB1 is a core component of the SWI/SNF chromatin remodeling complex, which regulates gene expression by modifying chromatin structure [45]. Additional genes implicated in HNS include *CTNNA3*, encoding a key component of adherence junctions essential for cell–cell adhesion, and *KMT2A,* which encodes a histone methyltransferase involved in the regulation of gene expression [3, 9]. Created in BioRender. Scheffler, J. (2025); agreement number: FH28JQKV2K https://BioRender.com/c0ub6nv

Full or partial loss of chromosome 22q can result in deletion of several tumour suppressor genes. The functions of genes altered in HNS overlap in the control of downstream cell signalling pathways (Fig. 5). Thus, activating mutations in *ERBB2* lead to constitutive activation of proliferative and anti-apoptotic cell signalling pathways such as the MAPK or PI3K signalling pathway [8, 42], as does loss of Merlin or LTZR1 protein [43, 44]. Like Merlin, CTNNA3 also inhibits the Hippo-YAP-associated protein/transcriptional coactivator with PDZ-binding motif (YAP/TAZ) signaling pathway. Loss of these proteins therefore leads to activation of YAP/TAZ and the downstream molecular processes contributing to tumour proliferation and EMT. Loss of KTM2A or SMARCB1 disrupts transcriptional regulation within the cells, potentially contributing to the aberrant gene expression profiles observed in tumour cells. In summary, the previously identified molecular features of HNS appear to converge functionally, resulting in a similar phenotype characterized by the activation of cellular and molecular alterations, as schematically shown in Fig. 5.

Take home messages

- Molecular analyses report that HNS can occur in NF2-, SMARCB1, LZTR1- related and NEC-SWN with proven germline *NF2* and *LZTR1* mutations.
- HNS show a methylation profile clustering to a subtype of Sw.
- Recurrent somatic findings of HNS include 22q loss, variants of *ERBB2* and *KMT2A*, and CTNNA3 loss.

### Artificial intelligence-based diagnosis of hybrid neurofibroma/schwannoma

To date, no study has used artificial intelligence (AI) to achieve a safe differential diagnosis of HNS from typical Sw using histological slides or radiological images. However, radiomics is very advanced in using AI, and radiomics-based machine learning models have demonstrated high accuracy in distinguishing Sw and Nf on MRI images in a multicentre study: logistic regression and support vector machine (SVM) classifiers achieved an area under the curve (AUC) of approximately 0.92 (accuracy ~ 92%) and thus significantly outperformed experienced examiners [46]. In a retrospective feasibility study with a medium-sized dataset (539 training and 94 validation cases), a pre-trained 2D convolutional neural network (CNN) (ResNet-34) detected vestibular Sw on individual contrast-enhanced MRI slices with high accuracy (internal validation 94.9%, external testing 91.2%) [47]. In addition, in a large retrospective cohort (861 VS patients, 1290 MRI studies), a deep learning-based 3D volumetry method (CNN-based, comparable to a 2.5D U-Net architecture) was established, in which the automatically determined tumour volumes after radiosurgery deviated by only ~ 1% from the manual reference measurements [48]. It is only a matter of time before an application is developed to distinguish HNS safely using radiomics and in pathology. Thus, Borji et al. have proven that classifying osteosarcoma histological whole-slide images into four categories (non-tumor, non-viable or viable tumor, and non-viable ratio) is superior to humans, with a performance of 99.08% accuracy, 99.10% precision, 99.28% recall, and a 99.23% F1 score [49]. Redirecting this idea to the differentiation of HNS histologically by using different categories, one for Nf and one for Sw, might hold enormous potential. However, to achieve such a promising model, a solid dataset needs to be gathered with as accurate ground truth as possible.

Take-home messages

- Whole-slide analysis of tumour specimens is rapidly developing in pathology and will be available for nerve sheath tumours in SWN.
- As the use of AI for whole-slide analyses has shown a precision better than human performance, AI might provide very promising tools for the safe diagnosis of rare HNS in the future.
- Nevertheless, the datasets of HNS that can be used to train AI are very small, and this still poses a challenge for the accurate conventional diagnosis of HNS.

## Conclusions and future directions

The distinction between HNS, Nf and Sw remains challenging and can result in the misdiagnosis of a neurofibroma suggesting NF1 instead of SWN. More germline and somatic pathogenic variants in SWN-associated HNS are being identified, and they comprise multiple genetic steps in 22q-deleted tumours and several possibilities of somatic events. Thus, the accuracy of molecular diagnostics for this primarily benign hybrid peripheral nerve sheath tumour is improving, providing a foundation for future personalized treatment strategies. In addition, AI-assisted image analysis holds promise for enhancing diagnostic accuracy and facilitating more refined subtyping of SWN-associated tumours.

## Data Availability

No datasets were generated or analysed.
